# P4HA2 promotes tumor progression and is transcriptionally regulated by SP1 in colorectal cancer

**DOI:** 10.1080/15384047.2024.2361594

**Published:** 2024-06-10

**Authors:** Xuening Dang, Xiaojian Chen, Zhonglin Liang, Zhujiang Dai, Wenjun Ding, Jinglue Song, Jihong Fu

**Affiliations:** a Department of Colorectal and Anal Surgery, Xinhua Hospital, Shanghai Jiaotong University School of Medicine, Shanghai, China; b Shanghai Colorectal Cancer Research Center, Shanghai, China; c Department of Cardiovascular Surgery, Shanghai Chest Hospital, Shanghai Jiaotong University School of Medicine, Shanghai, China

**Keywords:** P4HA2, colorectal cancer, EMT, AGO1, SP1

## Abstract

P4HA2 has been implicated in various malignant tumors; however, its expression and functional role in colorectal cancer (CRC) remain poorly elucidated. This study aims to investigate the involvement of P4HA2 in CRC metastasis and progression, uncovering the underlying mechanisms. In colorectal cancer (CRC), P4HA2 exhibited overexpression, and elevated levels of P4HA2 expression were associated with an unfavorable prognosis. Functional assays demonstrated P4HA2‘s regulation of cell proliferation, and epithelial–mesenchymal transition (EMT) both in vitro and in vivo. Additionally, the AGO1 expression was correlated with P4HA2, and depletion of AGO1 reversed the proliferation and EMT function induced by P4HA2. Chromatin immunoprecipitation (ChIP) and luciferase assays suggested that the transcription factor SP1 binds to the promoter sequence of P4HA2, activating its expression in CRC. This study unveiled SP1 as a transcriptional regulator of P4HA2 in CRC and AGO1 is a probable target of P4HA2. In conclusion, P4HA2 emerges as a potential prognostic biomarker and promising therapeutic target in colorectal cancer.

## Introduction

Colorectal cancer is one of the most common cancers world-wide and ranks as the fourth-leading cause of mortality among common cancers. The primary cause of cancer-related death in CRC was distant metastasis, which was linked to an adverse prognosis. Consequently, there is a significant unmet medical need for the prevention and treatment of progression and metastasis.

The molecular mechanism of progression and invasion of CRC remains to be clarified. Remodeling of extracellular matrix (ECM) was one of the hallmarks of tumor. Collagen is the most significant component of the ECM and the most abundant protein in human tissue.^1^ Synthesis of collage was a multi-step process and required collagen prolyl 4-hydroxylase (P4H). Collagen prolyl 4-hydroxylase (P4H) is an α2β2 tetrameric α-ketoglutarate (α-KG)-dependent dioxygenase that catalyzes 4-hydroxylation of proline to promote the formation of the collagen triple helix, releasing succinate as a product.^2^ Increased expression of P4HA2 was observed in various malignant tumors, including breast cancer, cervical cancer, and hepatocellular carcinoma.^3–5^ However, the expression of P4HA2 in colorectal cancer and the exact role that P4HA2 plays in colorectal cancer progression and metastasis and its underlying mechanisms remains unknown.

In the current study, we detected the expression of P4HA2 in colorectal cancer samples and found that P4HA2 was over-expressed in CRC tissues, the higher P4HA2 expression level was related with unfavorable prognosis of CRC patients. Furthermore, our study revealed that P4HA2 actively participate in regulating cell proliferation, migration, and EMT. AGO1 mediates the effects of P4HA2 on promoting colorectal cancer progression. Transcriptional factor SP1 could bind to the promoter sequence of P4HA2, thereby activating P4HA2 expression in the CRC. Hence, P4HA2 has the potential to serve as a prognostic biomarker and an encouraging therapeutic target.

## Results

### P4HA2 expression is correlated with tumor progression and poor prognosis

**Figure 1.**
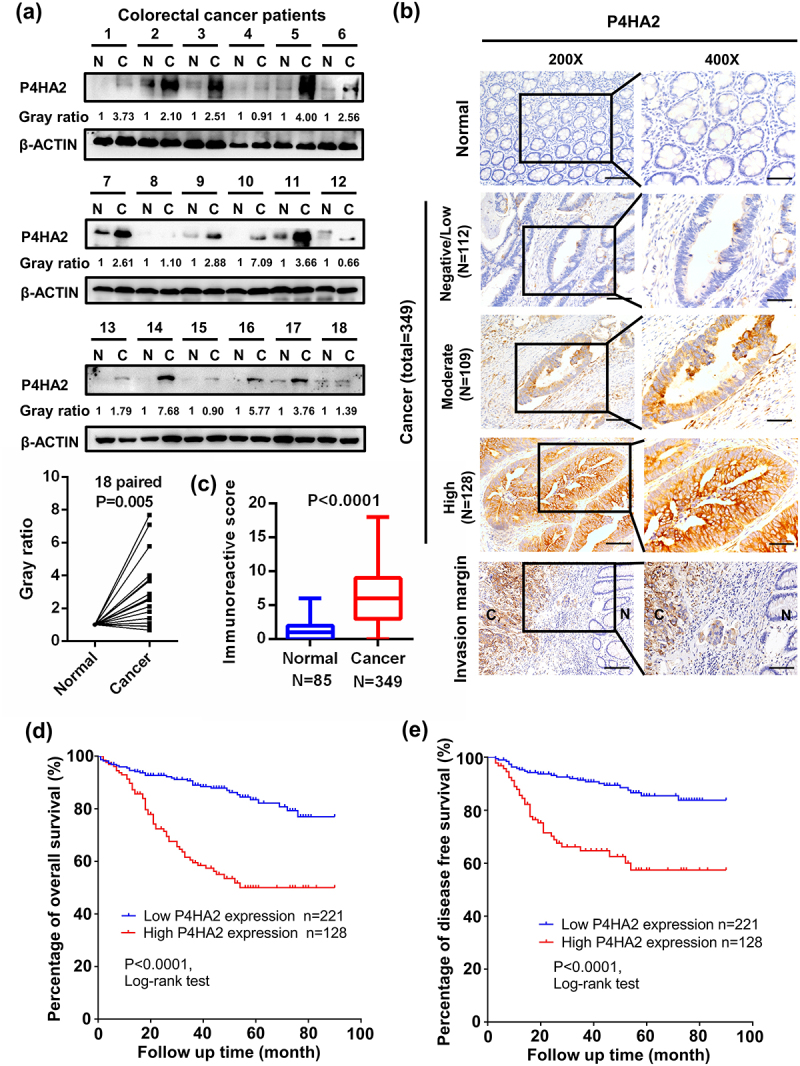
P4HA2 expression is upregulated in CRC tumor tissues and related bad clinical outcomes.

The TCGA database was employed to analyze the gene expression of P4HA2 in CRC tissues and normal tissues. We found that mRNA expression of P4HA2 was significantly higher in colorectal cancer than in normal tissues (Fig S1a). The protein expression of P4HA2 was subsequently analyzed in 18 colorectal patients, each paired with cancer and normal tissues were collected from the same patient. The expression level of P4HA2 protein was obviously higher in the CRC specimen compared with normal colorectal tissues (Figure 1a).

(a) A total of 18 pairs CRC tumor and paired normal tissues were used to detect P4HA2 expression by western blotting. Paired t-test was used for statistical analysis. (b) Immunohistochemical analysis of different P4HA2 expression in normal and tumor tissues, and tumor invasion margin. Representative images were shown (magnification: upper panels, 200×; lower panels, 400×). (c) The Mann–Whitney test was used to test the statistical significance of immunoreactive score. (d, e) Kaplan–Meier plots were stratified by P4HA2 protein expression level for overall survival (d) and disease-free survival(e) in CRC patients. Log-rank test was performed to assess the statistical significance.

We further investigated the expression level of P4HA2 in tissue microarray (TMA) composed by 349 CRC tissues and adjacent tumor colorectal tissues. Figure 1b showed typical images of different P4HA2 expression in normal and tumor tissues. The expression of P4HA2 was significantly higher in CRC cancer than in paired adjacent normal tissue (Figure 1b,c). The detailed demographic and clinical characteristics of the 349 CRC patients was shown in Table 1. To explore whether P4HA2 was capable of influencing the patho-clinical features of colorectal cancer, the expression of P4HA2 in CRC samples was classified into two groups according to the immunoactive score. Two hundred and twenty-one samples (63.3%) were assigned into high-level group and 128 samples (36.7%) into low-level group. Co-relation analysis with clinical pathological characteristics indicted that high expression of P4HA2 was correlated with N stage and M stage (Table 2).

**Table 1.** Baseline characteristics of patients.

Patient demographics (*N* = 349)	
Characteristics	N(%)
Age (years, mean ± s.d. (range))	64.5 ± 13.3（14–90）
Sex	
Male	198(56.7)
Female	151(43.3)
Follow‐up (month, mean ± s.d. (range))	44.5 ± 23.9(0–90)
Pathological type	
Adenocarcinoma	128(36.7)
Mucinous adenocarcinoma	203(58.2)
Signet-ring cell carcinoma	18(5.2)
Tumor differentiation	
Well	6(1.7)
Moderate	289(82.8)
Poor	54(15.5)
Location	
Right colon	73(20.9)
Transverse colon	18(5.2)
Left colon	25(7.2)
Sigmoid colon	74(21.2)
Rectum	159(45.6)
T-stage	
T1	8(2.3)
T2	33(9.5)
T3	128(36.7)
T4	180(51.6)
N-stage	
N0	191(54.7)
N1	107(30.7)
N2	51(14.6)
M-stage	
M0	303(86.8)
M1	46(13.2)
P4HA2 expression	
Low	221(63.3)
High	128(36.7)

**Table 2.** Comparison between P4HA2 high and low cases.

Characteristics	Low (*n* = 221)	High (*n* = 128)	
	N(%)	N(%)	*P*-value
Age (years,mean±s.d.)	64.4 ± 13.2	64.8 ± 13.5	.398
Sex			.302
M	130(58.8)	68(53.1)	
F	91(41.2)	60(46.9)	
Pathological type			.488
Adenocarcinoma	76(34.4)	52(40.6)	
Mucinous adenocarcinoma	134(60.6)	69(53.9)	
Signet-ring cell carcinoma	11(5.0)	7(5.5)	
Tumor differentiation			.461
Well	5(2.3)	1(0.8)	
Moderate	178(80.5)	111(86.7)	
Poor	38(17.2)	16(12.5)	
Location			.546
Right colon	46(20.8)	27(21.1)	
Transverse colon	12(5.4)	6(4.7)	
Left colon	14(6.3)	11(8.6)	
Sigmoid colon	41(18.6)	33(25.8)	
Rectum	108(48.9)	51(39.8)	
T-stage			.29
T1-T2	29(13.1)	12(43.0)	
T3-T4	192(86.9)	116(57.0)	
N-stage			<.001
N0	136(61.5)	55(43.0)	
N1-N2	85(38.5)	73(57.0)	
M-stage			<.001
M0	206(93.2)	97(75.8)	
M1	15(6.8)	31(24.2)	

To investigate the effect of P4HA2 on CRC patients, Kaplan–Meier analysis was performed. The results revealed that patients with high P4HA2 expression had significant worse overall survival (OS) and disease-free survival (DFS). (Figure 1d,e)

Furthermore, univariate analysis revealed that P4HA2 was associated with OS and DFS in CRC patients. Multivariate Cox-regression hazard analysis indicated that P4HA2 were independent prognostic factors for OS and DFS (Table 3).

**Table 3.** Relationship between clinicopathological characteristics and OS/DFS.

Variables	DFS				OS			
	Univariate analysis		Multivariate analysis		Univariate analysis		Multivariate analysis	
	Hazard ratio	*P*-value	Hazard ratio	*P*-value	Hazard ratio	*P*-value	Hazard ratio	*P*-value
Age								
<65	1				1			
≥65	1.66(1.06–2.60)	.057			1.67(1.09–2.566)	.079		
Sex								
Male	1				1			
Female	0.83(0.53–1.30)	.414			0.84(0.55–1.29)	.429		
Pathological type								
Adenocarcinoma	1				1			
Mucinous adenocarcinoma	2.16(0.52–8.90)	.286			1.06(0.38–2.96)	.907		
Signet-ring cell carcinoma	2.26(0.54–9.45)	.263			1.37(0.49–3.84)	.555		
Tumor differentiation								
Well	1				1			
Moderate	2.24(0.29–17.05)	.436			2.52(0.33–19.05)	.369		
Poor	1.92(0.27–13.83)	.519			2.02(0.28–14.51)	.487		
Location								
Right colon	1				1			
Transverse colon	1.52(0.87–2.65)	.139			1.37(0.80–2.34)	.254		
Left colon	1.37(0.53–3.52)	.515			1.13(0.45–2.88)	.794		
Sigmoid colon	1.31(0.58–2.98)	.518			1.16(0.52–2.60)	.719		
Rectum	1.09(0.59–1.99)	.787			1.07(0.61–1.87)	.825		
T-stage			1.79(1.11–2.88)	.017			1.74(1.10–2.74)	.017
Limited under serosa(T1–T3)	1				1			
Penetrating the serosa(T4)	2.24(1.40–3.58)	.001			2.23(1.42–3.49)	<.001		
N-stage			1.15(0.42–3.12)	.787			0.75(0.30–1.87)	.543
N0	1				1			
N1-N2	2.74(1.73–4.35)	<.001			2.59(1.67–4.01)	<.001		
M-stage			4.12(2.32–7.32)	<.001			2.92(1.71–5.00)	<.001
M0	1				1			
M1	6.86(4.27–11.02)	<.001			5.79(3.71–9.04)	<.001		
P4HA2 expression			2.28(1.42–3.66)	.001			2.46(1.56–3.88)	<.001
Low	1				1			
High	3.46(2.21–5.41)	<.001			3.69(2.39–5.68)	<.001		

### P4HA2 promotes CRC cell proliferation in vivo and in vitro

**Figure 2.**
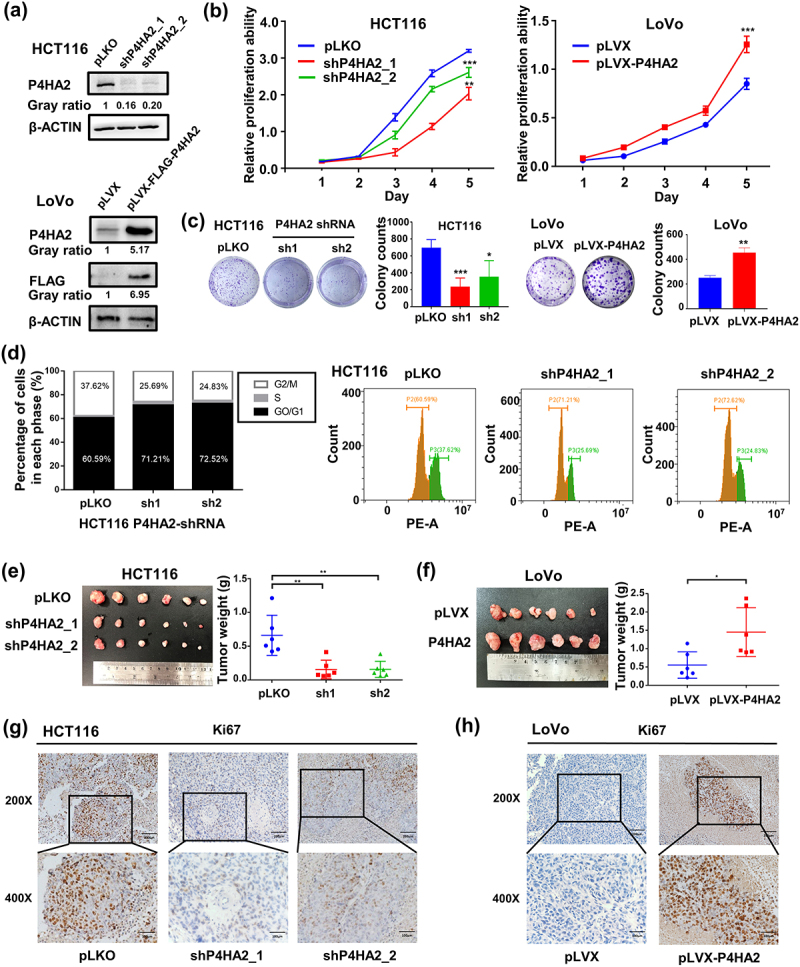
P4HA2 promotes CRC cell proliferation by regulating cell cycle in vivo and in vitro.

The mRNA and protein expression levels were detected in nine CRC cell lines and two normal colonic epithelial cell lines by qRT-PCR (Fig S1b) and western blot analysis (Fig S1c). To access the impact of P4HA2 on cell growth, proliferation, and cell cycle regulation, we established stably transfected P4HA2-knockdown CRC cell line using HCT116, in which the expression of P4HA2 is relatively high and chose relatively P4HA2 low expressed LoVo cells to establish the P4HA2-overexpressing cell line for further investigation. qRT-PCR and Western blot were performed for validation of the efficiency of knockdown and overexpression (Figure 2a and Fig S1d-f).

(a) The efficiency of P4HA2 stable knockdown in HCT116 and overexpression in LoVo was detected by western blotting. (b) CCK-8 assay was performed in the indicated HCT116 and LoVo stable cells to detect cell proliferation ability. (c) Colony formation assay in the indicated HCT116 and LoVo stable cells. (d) The percentage of HCT116 stable cells in each cell cycle phase was examined by cell flow cytometry analysis. Two kinds of HCT116-shP4HA2 cells, HCT116-pLKO cells, LoVo-pLVX cells and LoVo-P4HA2 cells were subcutaneous inoculated into nude mice to generate cell derived xenograft model. The mice were randomized, separated into five groups, and injected the above three different cells for 14 days. (e, f) Images of tumor from all groups after injection and the weight of the tumors from the different groups. (g, h) IHC assay was performed to stain KI67 in CDXs from the different groups. Representative images were shown (magnification: upper panels, 200×; lower panels, 400×). *p <.05, **p <.01, ***p <.001, ****p <.0001 by Student’s t-test.

The CCK-8 assay revealed that, compared with control groups, P4HA2 knockdown (KD) obviously inhibited the proliferation ability of HCT116 and LoVo (Figure 2b and S1g), while ectopic expression of P4HA2 promoted cell growth in LoVo cells (Figure 2b). Colony formation assay indicated the P4HA2 knockdown inhibited the colony formation and P4HA2 overexpression enhanced the colony formation (Figure 2c and Fig S1h).

Flow cytometry was performed to clarify whether P4HA2 affected the cell cycle regulation. As shown in Figure 2d, the cell cycle was obviously arrested. The percentage of G0/G1 phase cell was increased while the percentage of G2/M phase cell was decreased in P4HA2 depletion HCT116 cells (Figure 2d). Taken together, the results suggested that P4HA2 enhanced the proliferation of CRC cell line via regulation of cell cycle.

To explore the in vivo effect of P4HA2 on CRC cell proliferation, CRC cell-derived xenograft models were established. We injected subcutaneously the HCT116-shP4HA2 cells or LoVo-P4HA2 cells into nude mice. P4HA2 depletion obviously suppressed tumor growth (Figure 2e), whereas P4HA2 overexpression enhanced tumor growth (Figure 2f). The weight of the tumors yielded from the P4HA2 depletion group was lower than the control group while P4HA2 overexpression result in weightier tumor (Figure 2e,f). The expression of P4HA2 in P4HA2 knockdown and overexpression tumor were validated by western blotting (Fig S1i, S1j). Moreover, the expression level of proliferation marker, Ki67 was increased in P4HA2 overexpression tumors (Figure 2g) and decreased in P4HA2 knockdown tumors (Figure 2h). Thus, the results suggested that P4HA2 might promote CRC cell proliferation by regulating cell cycle.

### P4HA2 facilitates tumor migration by inducing EMT

**Figure 3.**
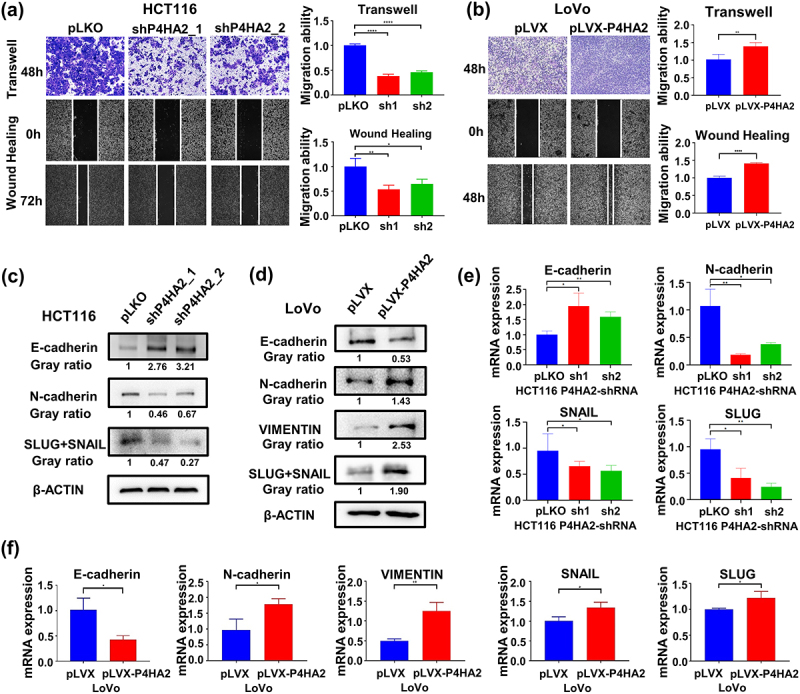
P4HA2 facilitates tumor migration by inducing EMT.

Wound-healing and transwell assay was performed to evaluate the effect of P4HA2 on the migration potential of CRC cells. P4HA2 KD impaired the mobility of HCT116 cells and LoVo cells compared with control groups (Figure 3a and Fig S1k), while P4HA2 overexpression elicited opposing effects (Figure 3b). These identical results indicated that P4HA2 participated in the regulation of CRC cells migration.

(a) Transwell and wound-healing assays were performed in P4HA2-knockdown and control HCT116 cells to determine the migration ability. (magnification: × 40). (b) Transwell and wound-healing assays were performed in P4HA2-overexpression and control LoVo cells to determine the migration ability. (magnification: × 40) (c, e) Mesenchymal hallmarks N-cadherin and SLUG+SNAIL as well as epithelial hallmark E-cadherin were detected in P4HA2 knockdown HCT116 cells by western blotting (c) and qRT-PCR (e). (d, f) Mesenchymal hallmarks N-cadherin, SLUG+SNAIL and VIMENTIN as well as epithelial hallmark E-cadherin were detected in P4HA2 overexpression LoVo cells by western blotting (d) and qRT-PCR (f). *p<.05, **p<.01, ****p<.0001 by Student’s t-test.

To determine whether P4HA2 affect migration of CRC cells via inducing EMT. Several EMT-related makers were measured by Western blot (Figure 3c,d) and qRT-PCR (Figure 3e,f). As Figures 3c & 3e shown, N-cadherin and SLUG/SNAIL expression were decreased, and E-cadherin expression was upregulated in both protein and mRNA level when P4HA2 knockdown. Meanwhile, as expected, N-cadherin, SLUG/SNAIL and VIMENTIN expression were increased, and E-cadherin expression was downregulated in both protein and mRNA level when P4HA2 overexpression (Figures 3d and 3f). Meanwhile, the cell morphology changes by the altered P4HA2 expression were shown in Fig S1l.

### AGO1 mediates the effects of P4HA2 on promoting colorectal cancer progression

**Figure 4.**
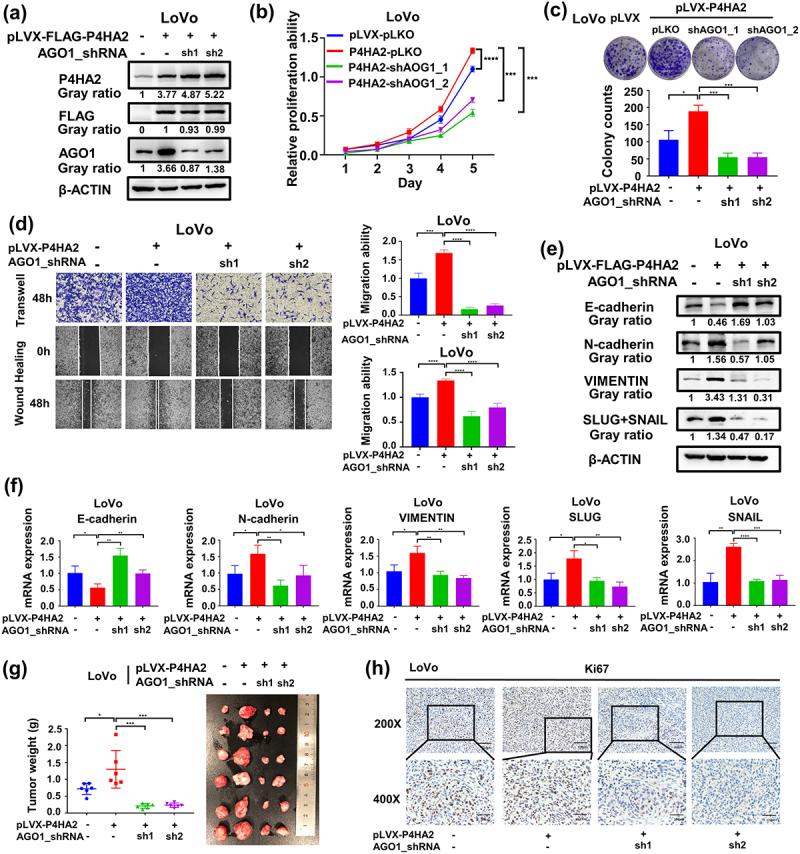
AGO1 mediates the effects of P4HA2 on promoting colorectal cancer progression.

To further elucidate the underlying molecular mechanism of the oncogenic role of P4HA2 in CRC. We aimed to investigate the potential regulatory effect of P4HA2 on AGO family which was reported in U2OS cells. The AGO expression was detected in P4HA2 KD HCT116 cells. AGO1 was decreased in P4HA2 KD CRC cells (Fig S2a) while, AGO2/3/4 expression did not change (Fig S2b). Consistently in the TCGA CRC dataset, the mRNA level of P4HA2 was positively correlated with the AGO1 mRNA level (Fig S2c). Moreover, upregulated P4HA2 expression led to increased AGO1 mRNA and protein expression in CRC cells (Fig S2d). To verify whether P4HA2-induced tumor progression was achieved in an AGO1-dependent manner. Colorectal cancer cell LoVo with stably expression P4HA2 and knockdown AGO1 was constructed (Fig S2e and 4a). CCK-8 assay (Figure 4b) and colony formation assay (Figure 4c) detected that the proliferation ability promoted by P4HA2 overexpression was abolished by AGO1 knockdown. Transwell and wound-healing assay were performed, as results of the assays, we found that the increase in migration of LoVo cells seen with P4HA2 overexpression was rescued by AGO1 knockdown (Figure 4d). EMT hall-markers were consequently detected by western blotting and qRT-PCR. Knockdown AGO1 reversed the changes of EMT hallmarks caused by overexpression of P4HA2 (Figure 4e–f).

(a) The expression of P4HA2 and AGO1 were detected in the indicated stable LoVo cells by western blotting. (b, c) CCK-8 and colony formation assays were performed in the indicated LoVo stable cells to detect cell proliferation ability. (d) Transwell and wound-healing assays were performed in the indicated LoVo stable cells to determine the migration ability. (magnification: × 40). (e, f) Mesenchymal hallmarks N-cadherin, SLUG+SNAIL and VIMENTIN as well as epithelial hallmark E-cadherin were detected in the indicated LoVo stable cells by western blotting (e) and qRT-PCR (f). (g) Images of tumor from all groups after injecting the indicated LoVo stable cells. The weight of the tumors from the four groups. (h) IHC assay was performed to stain KI67 in CDXs from the four groups. Representative images were shown (magnification: upper panels, 200×; lower panels, 400×). Pearson correlation analysis was used to evaluate the associations. *p<.05, **p<.01, ***p<.001, ****p<.0001 by Student’s t-test.

Furthermore, to confirm the functional correlation between P4HA2 and AGO1 in CRCs in vivo. P4HA2-LoVo cell lines with/without AGO1 shRNA were injected subcutaneously into nude mice. AGO1 knockdown could restore the effect of P4HA2 on increasing tumor weight (Figure 4g). The expression of P4HA2 and AGO1 was validated by Western blot (Fig S2f). According to the IHC result, changes of KI67 expression in tumor tissues indicated that P4HA2 had a biological role in regulating tumor formation by regulating the expression of AGO1 in vivo (Figure 4h). Collectively, these data suggested that the effects of P4HA2 on promoting colorectal cancer progression were mediated by AGO1 in vitro and in vivo.

### SP1 transcriptionally activates P4HA2 gene expression in colorectal cancer

**Figure 5.**
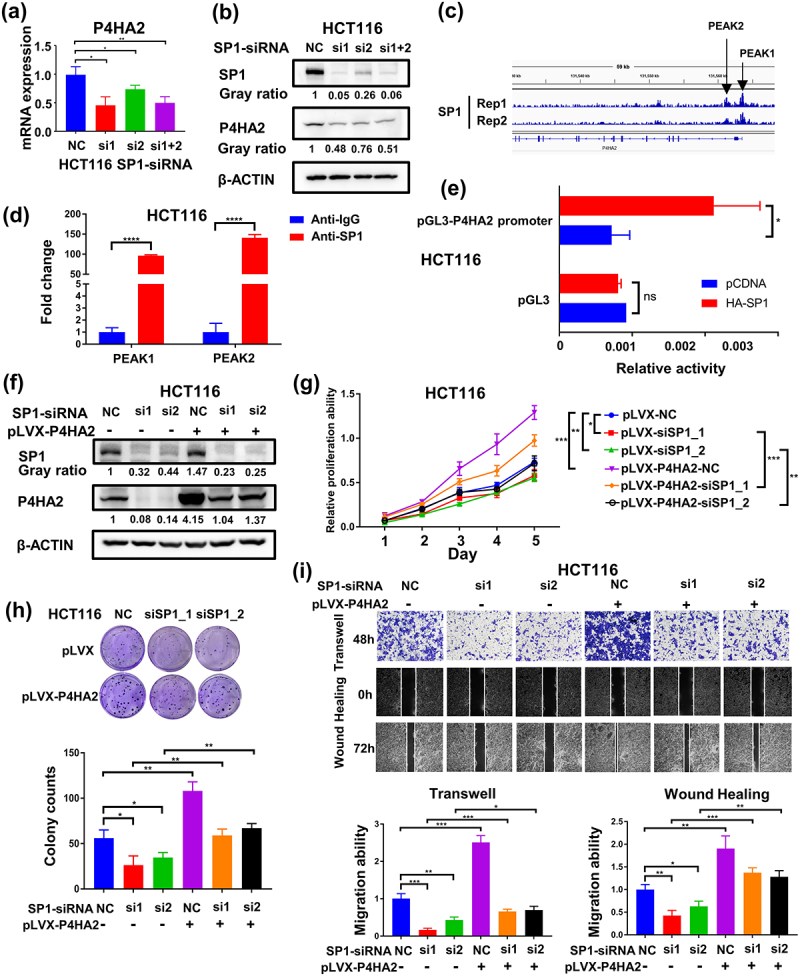
SP1 transcriptionally activates P4HA2 gene expression in colorectal cancer.

To explore the underlying mechanism of P4HA2 up-regulated in colorectal cancer, we objected to clarify whether the expression of P4HA2 was regulated by transcriptional factor. Using JASPAR database, we found that there was 20 SP1 binding sites in the promoter region of P4HA2. Therefore, we further validated the relationship between the expression of SP1 and P4HA2 by analyzing the TCGA database. We found that the level of SP1 mRNA was positively correlated with the level of P4HA2 (Fig S2g). The SP1 knockdown dramatically decreased the P4HA2 expression levels in CRC cell lines (Figure 5a-b and S2h). By analyzing the ChIP-seq data of SP1 in HCT116 cells, we found that there were two binding peaks between SP1 and P4HA2 genes (Figure 5c). Consequently, the P4HA2 promoter region including binding sites of SP1 with a high score was inserted into pGL3 vector, and dual-luciferase reporter assay indicated that SP1 significantly activated the transcription of P4HA2 (Figure 5d). To verify whether SP1 activated P4HA2 directly, Chip-qPCR assay was performed. We designed primers for two binding peaks of P4HA2 promoter, and the ChIP-qPCR results revealed that SP1 could bind to both two regions of P4HA2 (PEAK 1: −0.3 kb, PEAK 2: +0.3 kb) (Fig S2i), implicating the direct transcriptional regulation of P4HA2 by SP1 (Figure 5e).

(a) The expression of P4HA2 were detected in the SP1 knockdown HCT116 cells by qRT-PCR. (b) The expression of P4HA2 and SP1 were detected in the SP1 knockdown HCT116 cells by western blotting. (c) SP1 binds to the P4HA2 gene locus in two different HCT116 ChIP-seq data sets. (d) Luciferase assay was performed to detect the activation of P4HA2 gene expression by SP1. The P4HA2 promoter regions used for luciferase assay was shown. (e) ChIP-qPCR analysis of SP1 binding to the P4HA2 gene locus in HCT116 cells. Rabbit IgG was used as the negative control. (f) The expression of P4HA2 and SP1 were detected in the indicated HCT116 cells by western blotting. (g, h) CCK-8 assay and colony formation were performed in the indicated HCT116 cells to detect cell proliferation ability. (i) Transwell and wound-healing assays were performed in the indicated HCT116 cells to determine the migration ability. (magnification: × 40). *p<.05, **p<.01, ***p<.001, ****p<.0001 by Student’s t-test.

To further confirm that the enhanced expression of P4HA2 mediates the tumor promotion function of SP1 in CRC, we rescued P4HA2 expression in SP1 knockdown CRC HCT116 cells by stably expressing P4HA2 (Figure 5f and S2j). Rescue of P4HA2 expression reversed the suppression of proliferation and migration abilities induced by knockdown of SP1 in HCT116 cells (Figure 5g–i)

In summary, we concluded that P4HA2 can promote the proliferation and migration abilities, which was transcriptionally regulated by SP1.

## Discussion

CRC is a common human malignancy, and the in-depth understanding of its molecular mechanism is urgently needed. In this study, we characterized the roles of P4HA2 in colorectal cancer. We highlighted that the elevated expression of P4HA2 was closely associated with unfavorable prognosis of CRC patients. We identified P4HA2 as an oncogene that promotes the proliferation, invasion, migration, and accelerating the cell cycle transition in CRC. Moreover, we found that P4HA2 promotes EMT in CRC. Mechanistically, transcriptional factor SP1 could directly bind to the promotor region of P4HA2 and activates the expression of P4HA2 in colorectal cancer and P4HA2 promotes CRC progression and EMT by inducing expression of AGO1.

The role of P4HA2 has been investigated in a series of cancers. P4HA2 protein levels were reported to be overexpressed in several malignancies, including breast cancer, hepatocellular carcinoma, and cervical cancer, and correlated with poor prognosis.^3–7^ Studies on breast cancers suggested that P4HA2 promotes tumor metastasis and progression via regulation of collagen deposition. In cervical cancer, P4HA2 induces EMT and cell glycolysis, however, the expression of P4HA2 in CRC and the concrete molecular mechanism of P4HA2 in CRC progression and metastasis are remained to be further explored.

Herein, in agreement with the previous studies, we demonstrated that P4HA2 might be an important regulator in CRC. First, Western blot assay using clinical samples from 18 CRC tissues revealed that P4HA2 protein was overexpressed in the CRC specimen compared with normal colorectal tissues. Second, IHC staining of P4HA2 in TMA containing 349 CRC tissues showed P4HA2 expression was higher in CRC tissues. Furthermore, Kaplan–Meier survival analysis revealed that patients with higher expression of P4AH2 in CRC tissues had significant worse overall survival and disease-free survival. Multivariate Cox-regression hazard analysis indicated that P4HA2 were independent prognostic factors for OS and DFS. These results suggested the critical role of P4HA2 in colorectal cancer.

The mechanism of oncogenic role of P4HA2 was investigated in a series of tumors. In melanoma, P4HA2 promotes tumor proliferation and invasion. In breast cancer and liver cancer, P4HA2 modulates collagen deposition in ECM and promotes tumor progression and metastasis. In cervical cancer, P4HA2 was reported to regulate cell metabolism and EMT. In this study, the further in vitro and in vivo assays indicated P4HA2 promote tumor proliferation, invasion, and migration through modulating cell cycle and EMT.

EMT allows the solid tumors to become more malignant, enhancing tumor invasiveness and metastatic activity.^8^ During the EMT process, cells undergo phenotypic changes and molecular alterations.^9^ Cancer cells losing epithelial markers such as E-cadherin, and gaining mesenchymal markers, such as vimentin, N-cadherin, and SNAIL.^10^ Thus, the expression level of the EMT markers could reveal the extent of EMT. In this study, we found that knockdown of P4HA2 in CRC cells decreased the expression level of N-cadherin and SLUG/SNAIL, and enhanced the expression of E-cadherin. These findings suggested that P4HA2 has the capability to induce EMT in CRC cells.

Less attention has been paid to the molecular mechanism of elevated P4HA2 expression in malignancies. Previous studies indicate expression of P4HA2 was regulated by miRNAs. In aspirin treated hepatocellular carcinoma model, P4HA2 expression was inhibited through axis of NF-kB/P4HA2 and LMCD1-AS1/let-7 g/P4HA2.^11^ Another research found that HBV X protein was capable of inducing P4HA2 through suppression miR-30e, in which miR-30e could target P4HA2 mRNA 3’ untranslated region in liver cancer cells.^3^ Specificity protein 1 (SP1) is a transcriptional factor that has been shown to be overexpressed in various malignancies and is associated with poor prognosis and unfavorable clinical characteristics.^12–14^ SP1 may control genes implicated in tumor survival, progression, and metastasis.^15^ The present study revealed SP1 could bind to the promotor region of P4HA2 and promotes the P4HA2 transcription. The finding of our study provided a new mechanism on P4HA2 over expression in cancer cells.

The AGO family is a key component of RNA-induced silencing complex and was reported to play important in solid tumor.^16,17^ Hydroxylation of AGO2 protein at proline-700 by prolyl-4-hydroxylase enhanced the stability of AGO2 protein whereas depletion of P4HA1 or P4HB diminished the stability of AGO2 protein.^18^ Under hypoxia conditions, prolyl-4-hydroxylase was increased and led to prolyl hydroxylation and accumulation of AGO2.^19^ In CRC, the inhibition of P4HA1 reduced the expression level of AGO2 protein,^20^ suggesting AGO2 was the target of prolyl-4-hydroxylase. In this research, we hypothesized a correlation between AGO family and P4HA2 in colorectal cancer. The expression of AGO family was evaluated in P4HA2 knockout CRC cells, and we found that AGO1 was decreased in P4HA2 KD CRC cells, while AGO2/3/4 expression were not influenced by P4HA2 depletion. Furthermore, upregulated P4HA2 led to increase AGO1 mRNA and protein expression. The further in vitro assay indicates that enhancement of proliferation and migration by P4HA2 was achieved in an AGO1-dependent manner.

AGO1 has been identified as an oncogene in several malignancies.^21^ AGO1 expression was significantly higher in cancerous tissue than in adjacent tissue in colon cancer, and it may serve as a potential marker of tumor occurrence.^22^ In HCC cell, AGO1 may promote HCC metastasis via the TGF-β pathway,^17^ and in lung cancer, AGO1 promotes the proliferation and motility of cancer cells.^21^ The findings of our study suggest a correlation between AGO1 and P4HA2 expression levels and function in CRC cells. Compared with the results of previous study, suggesting post-translational modification of AGO by P4HA1,^18,19^ AGO1 expression was affected by P4HA2 in mRNA and protein level, the underlying mechanism would be elucidated in further study. Taken together, the present study provides the initial evidence of P4HA2 over-expression in CRC, suggesting its potential role as a prognostic marker. We revealed a novel mechanism for P4HA2 overexpression by demonstrating that P4HA2 was controlled by transcriptional factor SP1. Additionally, our investigation indicates AGO1 expression was correlated with P4HA2 and is a potential target of P4HA2.

Collectively, based on the findings in this research, we proposed targeting SP1-P4HA2-AGO1 axis might be a viable treatment approach in CRC.

## Methods and materials

### Patients and specimens collection

All human CRC and paired normal tissue samples from patients underwent surgery in the Department of Colorectal Surgery, Xin‐Hua Hospital, Shanghai Jiaotong University School of Medicine was collected. A total of 349 tissues were made into tissue microarray (TMA) for further immunohistochemistry (IHC) analysis of P4HA2. Another 18 paired tissues were collected for western blot analysis of P4HA2. The study was approved by the Ethics Committee of Xinhua Hospital

### IHC and histopathologic evaluation of P4HA2 expression

Experiments were performed as previously described.^23^ Details of primary antibody used in this study were presented in Supplementary Table S1. The P4HA2 expression in TMA was accessed and semi-quantitatively scored based on IHC results by two independent pathologists. And the final scores of P4HA2 expression in each CRC patients were calculated by multiplying the proportion score by the intensity score. The score standard of staining proportion was as follows: 0 (0%), 1 (>0 to ≤25%), 2 (>25 to ≤50%), 3 (>50 to ≤75%), and 4 (>75%). In addition, the staining intensity was scored from 0 to 3, indicating the negative, weakly positive, moderately positive, and strongly positive expression, respectively. Samples with a staining score of ≤6 were divided into the P4HA2 low‐expression group and those with a score of >6 were identified as the P4HA2 high‐expression group in this study, respectively.

### Cell culture

HEK293T cells, a total of nine human CRC cell lines including HCT116, LoVo, SW480, SW620, HT29, DLD1, RKO, LS174T, Caco2 cells, the normal colonic cell line CCD841 and NCM460 were purchased from the American Type Culture Collection (ATCC, Manassas, Virginia). These cells were maintained in Dulbecco’s modified Eagle’s medium (DMEM; HyClone, Los Angeles, CA) supplemented with 10% fetal bovine serum (FBS; Gibco, Grand Island, New York, NY), 1% penicillin/streptomycin at 37°C in 5% CO2.

### Transfection, vector construction, and virus production

Transient transfection was performed by using PEI (Polysciences) or Lipofectamine 2000 (Invitrogen) according to the published manufacturers’ protocols. Lipofectamine RNAiMAX (Invitrogen) was used for siRNA transfection. All siRNA oligonucleotides were synthesized by Shanghai Genepharma company. The siRNA sequence was listed in Supplementary Table S2. To knockdown P4HA2/AGO1 expression, the short hairpin RNA (shRNA) was performed. The shRNA oligonucleotides of P4HA2/AGO1 were cloned into the pLKO.1 (puro) vector. HCT116 and LoVo cells stably expressing shRNA were generated by infection of lentivirus, which was produced by HEK293T cells using envelope plasmid pMD2.G and packaging plasmid psPAX2. Polybrene was used to promote transfection efficiency and infected stable cell lines were selected by puromycin. The shRNA sequences were provided in Supplementary Table S2. Human P4HA2 cDNA was cloned into the pLVX (puro) vector. The primers for cloning were provided in Supplementary Table S2. Cell lines stably expressing P4HA2 cDNA were also generated by infection of lentivirus. The cells were infected as described.

### Western blotting and qRT-PCR assay

Western blotting and qRT-PCR experiments were performed as described.^24^ Briefly, for Western blotting assay, the protein was collected and quantified by Detergent Compatible Bradford Protein Assay Kit (Beyotime, Shanghai, China). The intensity of the protein bands was analyzed by enhanced chemiluminescence with anti‐β actin as control. And for qRT-PCR assay, total mRNA was isolated from CRC patients and different CRC cell lines by using RNAiso Plus Reagent (Takara Biotechnology Co., LTD., Dalian, China). MRNA was reversed by using the PrimeScript™ RT Master Mix (Takara Biotechnology LTD). SYBR Premix ExTaq™ (Takara, Japan) and Applied Biosystems 7500 Fast Real-Time PCR System (Applied Biosystems, Waltham, MA) were then used. The PCR reaction was repeated three times. The antibodies used and all qRT-PCR primer sequences used in this study were provided in Supplementary Table S1 and S2.

### Cell proliferation assay

Cell proliferation was assessed by using the CCK-8 kit (Dojindo Molecular Technologies, Tokyo, Japan). One thousand cells/wells were seeded in 96-well plates and incubated as aforesaid. After incubation for 24 hours, 10 μL CCK-8 solution was added into each cell incubator for 1.5 hours in the dark conditions. For the following 5 days, the plates were measured by detecting the absorbance at 450 nm in a microplate reader after CCK-8 treated.

The colony formation assay was conducted to detect the ability of cell proliferation. One thousand cells/wells were counted and seeded in 6-well plates and cultured as aforesaid for 2 weeks. The cells were then washed by PBS, fixed with 4% paraformaldehyde (PFA) for 30 minutes and stained with 0.1% crystal violet at room temperature for 10 minutes. All experiments were performed in triplicate.

### Cell cycle detection

The cell cycle of HCT116 cells were detected by flow cytometry. The experiments were performed as described.^24^ Briefly, 2 × 10^5^ cells were harvested and washed once with cold PBS. Seventy-five percent pre-iced ethanol was used for 30-min fixation, after centrifugated at 200 g and supernatant discarded. Propidium iodide (PI, 20 μg/ml) was then used to stain the indicated cells for 30 min at 4°C avoiding light. In the end, flow cytometry (Beckman Coulter, Brea, CA) with the CytExpert Software (version no. 1.2.11.0; Beckman Coulter) was used to detect the HCT116 cell cycle.

### Transwell and wound-healing assay

These experiments were performed to assess cell migration ability. For transwell assay, 1 × 10^5^ cells were counted and seeded into an 100 μl FBS-deficient upper chamber, while the lower chamber was added with 600 μl complete medium and incubated for 48 hours. The upper chamber was then washed by PBS, fixed with 4% paraformaldehyde (PFA) and stained with 0.1% crystal violet at room temperature. The number of cells that penetrated the upper chamber were counted and the images were shot.

For wound-healing assay, 1 × 10^6^ cells/well were counted and seeded into six-well plates and cultured as aforesaid. A scratch wound was made in the cell monolayer and the initial image should be immediately shot when the cell density up to 100%. Then the cells were cultured with low-serum medium (DMEM containing 1% FBS) for another 72 or 48 hours and the corresponding images were shot. All experiments were performed in triplicate.

### Luciferase reporter assay

The P4HA2 promoter (−2000bp) was cloned into the pGL3-basic luciferase reporter vector. Cells were co-transfected with pGL3-Vector/P4HA2 and the indicated plasmids, and the activities of firefly luciferase and Renilla luciferase were measured using a dual luciferase reporter assay (Promega). The relative reporter activity was normalized to the activity of Renilla luciferase.

### ChIP-qPCR assay

Chromatin immunoprecipitation (ChIP) assays were performed by using a chromatin immunoprecipitation kit (Millipore) according to the attached manufacturer’s instructions. All ChIP-qPCR primers are listed in Supplementary Table S2.

### Tumor formation assay in nude mice

The 6-week-old male nude mice obtained from SLAC Laboratory Animals LLC, Shanghai, China, were used for tumor formation assay. The nude mice were injected with indicated HCT116 and LoVo cells, a group of four nude mice were injected with one kind of cells. Each nude mouse was subcutaneously injected into both axillary fat pads with 1 × 10^6^ cells. After two weeks, the nude mice were killed and operated to isolate tumors. The tumors were weighed and sectioned for immunohistochemistry. All mouse experimental procedures were approved by the Laboratory Animal Care and Welfare Committee of Xinhua Hospital.

### Statistical analysis

Statistical analysis was performed using GraphPad Prism 5 Software (GraphPad, San Diego, CA) and SPSS version 19.0 software (IBM, 2010, Chicago, IL). The Kaplan–Meier method was performed to estimate OS and DFS among the different prognostic groups, and the log-rank test was also used. The Cox‐proportional hazard model was used to calculate the hazard ratio (HR) for multivariate survival analyses and the confidence intervals (CI) were set at 95%. Pearson correlation coefficients were used to evaluate the correlations between the mRNA expression of P4HA2 and that of AGO1 or SP1. Unpaired Student’s t-test was used to compare two different groups. All tests correspond to two-sided, and *p* values < .05 were considered statistically significant.

## Abbreviations

P4HA2: prolyl 4-hydroxylase subunit alpha-2
CRC: colorectal cancer
SP1: specificity protein 1
EMT: Epithelial-Mesenchymal Transition
AGO1: Argonaute RISC Component 1
ECM: extracellular matrix
α-KG: α-ketoglutarate
TMA: tissue microarray
IHC: immunohistochemistry
siRNA: Small interfering RNA
shRNA: short hairpin RNA
qRT-PCR: Quantitative Real-time PCR
CCK-8: cell counting kit‐8
PFA: paraformaldehyde
DMEM: Dulbecco’s Modified Eagle’s Medium
ChIP: Chromatin immunoprecipitation
HCC: hepatocellular carcinoma
TGF-β: Transforming growth factor beta

## Supplementary Material

Supplementary Table S2 .docx

Supplementary Table S1 .docx

## Data Availability

The datasets generated during and analyzed during the current study are available from the corresponding author on reasonable request
