# Supplementary material for: P4HA2 promotes tumor progression and is transcriptionally regulated by SP1 in colorectal cancer
Source: Cancer Biol Ther. 2024 Jun 10;25(1):2361594. doi: 10.1080/15384047.2024.2361594 (PMC11168210; doi:10.1080/15384047.2024.2361594)
Supplement: Supplementary Table S2 .docx [file KCBT_A_2361594_SM6623.docx]

**Supplementary Table S2 The primers and related sequences used in this study**

| **Primers and related sequence** | **Sequences (5'-3')** |
| --- | --- |
| **P4HA2** | **Forward: AGCTCAGGACACCAAACCAG** |
|  | **Reverse: CTTCCTGCCTCAGGTATTGC** |
| **Beta-actin** | **Forward：GCACAGAGCCTCGCCTT** |
|  | **Reverse：GCACAGAGCCTCGCCTT** |
| **E-cadherin** | **Forward: AAAGAGAGTGGAAGTGCCCG** |
|  | **Reverse: GCAGGTGGAGAACCATTGTC** |
| **N-cadherin** | **Forward: TCAGGCGTCTGTAGAGGCTT** |
|  | **Reverse: ATGCACATCCTTCGATAAGACTG** |
| **SLUG** | **Forward：GTGATTATTTCCCCGTATCTCTAT** |
|  | **Reverse：CAATGGCATGGGGGTCTGAAAG** |
| **SNAIL** | **Forward：TCGGAAGCCTAACTACAGCGA** |
|  | **Reverse：AGATGAGCATTGGCAGCGAG** |
| **AGO1** | **Forward:** **GGTCGAGAAGTGCCCTGAAT** |
|  | **Reverse：CGGTGGACAGAAGTGGGAAT** |
| **AGO2** | **Forward:** **GATCGGCAAGAAGAGATTA** |
|  | **Reverse：CGTCTTGCCGGCCAGGATGAC** |
| **AGO3** | **Forward:** **GGGCAAACCCATTAAACTGC** |
|  | **Reverse：AACTGGTCTACGGTCTCCAA** |
| **AGO4** | **Forward:** **CAGGAATTCAGGGAACCAGCCG** |
|  | **Reverse：CTGCCTTCCGCACTGTCATGATC** |
| **SP1** | **Forward: CCCAGGGCCCGAGTCAGTCA** |
|  | **Reverse：GGGAGCGGCAGCCACAACAT** |
| **pLVX-P4HA2 construction** | **Forward1:** **GAGCTGCGAGTGTCCAGA** |
|  | **Reverse1：TTCAGTCACACAGGAGTCGC** |
|  | **Forward2:** **AGCGTTGTTTTTCCTTGGCA** |
|  | **Reverse2: TTGACATGGGCTGAAGGACC** |
|  | **Forward3:TCTATTTCCGGTGAATTCC ATGGATTACAAGGATGACGACGATAAG AT GAAACTCTGG GTGTCTG** |
|  | **Reverse3:** **GGGAGGGAGAGGGGCGGGATCC TCAGTC AACTTCTGTT GATC** |
| **P4HA2 promoter construction** | **Forward1:** **GGAAGTAAGGAAAATCTCTCTGCC** |
|  | **Reverse1：CGTCGCCCGGTCAGC** |
|  | **Forward2:** **CTGAAGGTTTTAATCAGGGAGGG** |
|  | **Reverse2：CCCTTGGCGACTCCGC** |
|  | **Forward3: AACATTTCTC TATCGATAGGTACC ATGGCCAGTTAGAGAGACTG** |
|  | **Reverse3：TACCGGAATGCCAAGCTT CTGAAAGGCATTCAATGAC** |
| **siP4HA2_1** | **GCAGTCTCTGAAAGAGTACAT** |
| **siP4HA2_2** | **GCCGAATTCTTCACCTCTATT** |
| **siSP1_1** | **GCTGGTGGTGATGGAATACAT** |
| **siSP1_1** | **CCACTCCTTCAGCCCTTATTA** |
| **shP4HA2_1** | **Forward:CCGGGCAGTCTCTGAAAGAGTACATCTCGAGATGTACTCTTTCAGAGACTGCTTTTTG** |
|  | **Reverse:AATTCAAAAAGCAGTCTCTGAAAGAGTACATCTCGAGATGTACTCTTTCAGAGACTGC** |
| **shP4HA2_2** | **Forward:CCGGGCCGAATTCTTCACCTCTATTCTCGAGAATAGAGGTGAAGAATTCGGCTTTTTG** |
|  | **Reverse:AATTCAAAAAGCCGAATTCTTCACCTCTATTCTCGAGAATAGAGGTGAAGAATTCGGC** |
| **P4HA2 PEAK1** | **Forward:** **CAGAGGACGGCGAACCC** |
|  | **Reverse：TCCTCCCTTGGCGACTCC** |
| **P4HA2 PEAK2** | **Forward:** **GATCCGGGGCCGGGGGTCG** |
|  | **Reverse：GCAGAACGGGCTTAGCCAATA** |
