# Supplementary material for: P4HA2 promotes tumor progression and is transcriptionally regulated by SP1 in colorectal cancer
Source: Cancer Biol Ther. 2024 Jun 10;25(1):2361594. doi: 10.1080/15384047.2024.2361594 (PMC11168210; doi:10.1080/15384047.2024.2361594)
Supplement: Supplementary Table S1 .docx [file KCBT_A_2361594_SM6622.docx]

**Supplementary Table S1** List of the primary antibodies used in this study, and information on working dilutions of antibodies in Western blotting (WB) and immunohistochemistry (IHC).

| Antibody | Species | Source | Catalog# | Applications | Dilution |
| --- | --- | --- | --- | --- | --- |
| P4HA2 | Mouse | Abcam | ab211527 | WB  IHC | 1:1000  1:100 |
| N-cadherin | Mouse | BD Pharmingen™ | 740091 | WB | 1:1000 |
| E-cadherin | Mouse | BD Pharmingen™ | 743712 | WB | 1:1000 |
| SLUG+  SNAIL | Rabbit | Abcam | ab180714 | WB | 1:1000 |
| VIMENTIN | Rabbit | Cell Signaling Technology | #5741 | WB | 1:1000 |
| AGO1 | Rabbit | Cell Signaling Technology | #5053T | WB | 1:1000 |
| SP1 | Rabbit | Cell Signaling Technology | # 9389S | WB | 1:1000 |
| FLAG | Rabbit | Cell Signaling Technology | #14793 | WB | 1:1000 |
| Ki67 | Rabbit | Abcam | ab15580 | IHC | 1:100 |
| β-Actin | Rabbit | Abcam | ab8226 | WB | 1:10000 |
